# Advancement of methods in insect science: from genes to ecosystems

**DOI:** 10.1093/jisesa/ieag012

**Published:** 2026-03-02

**Authors:** Amr Mohamed, Wei Zhang, Samuel Ward, Natraj Krishnan

**Affiliations:** Department of Entomology, Faculty of Science, Cairo University, Giza, Egypt; State Key Laboratory of Green Pesticide, Guizhou University, Guiyang, China; Department of Entomology, The Ohio State University, Columbus, OH, USA; Laboratory of Insect Physiology and Biochemistry, Department of Agricultural Sciences and Plant Protection, Mississippi State University, Starkville, MS, USA

## Preface

Innovative advances in methods drive discovery in insect science. For example, the development of broadly conserved cytochrome c oxidase subunit I (COI) primers enabled widespread, comparable molecular identification across metazoans and directly catalyzed large-scale DNA barcoding and biodiversity-monitoring programs (Folmer et al. 1994). Leveraging this advancement, whole-genome sequencing of *Drosophila melanogaster* then translated those molecular resources into a methodological foundation for functional genetics and comparative genomics that has underpinned modern population- and evolutionary-genomic studies (Adams et al. 2000). Likewise, the application of systematic, network-level sampling and quantitative analysis revealed how interaction topology determines community vulnerability and thus showed that rigorous sampling designs and analytical methods are central to reliable ecological inference and conservation action (Memmott et al. 2004).

Methodological advances often arrive quietly—a new reagent here, a tweak to an imaging pipeline there, a re-purposing of a technology already available, to gain more insights into a physiological system—but together, they reshape what we can measure, how accurately we measure it, and who can take those measurements. This special collection gathers 7 such advances that are united by a pragmatic ambition: to make insect science more precise, less invasive, more reproducible, and more affordable so insights can scale from museum cabinets to landscapes and from laboratories to management programs. The articles span chemical marking, automated image analysis, metabarcoding, nondestructive DNA extraction, low-cost tracking, thermal physiology, and deep-learning bioacoustics—a deliberately interdisciplinary set chosen for clarity of validation and potential for broad uptake.

## Precision, Accessibility, and the Limitation of Trade-Offs

A clear throughline in the collection is attention to operational constraints. Faraji et al. (2025) tackle a classic problem for public-health programs: how to produce reliable mosquito counts at scale. Their ImageJ-based optical counting pipeline maintains <5% error across seasons and sample conditions, and—importantly—does so without frequent recalibration, offering a realistic path to consistent longitudinal surveillance (Faraji et al. 2025). By contrast, Liu et al. (2025) evaluate an external fluorophore marker for navel orangeworm and show how pragmatic choices—color, dose, and detection method—change the balance between detectability and logistical simplicity; green and yellow markers and visual UV inspection provided durable, field-friendly solutions (Liu et al. 2025). Both studies emphasize that robustness under realistic field and operational constraints is as important as theoretical sensitivity.

However, robustness often comes with trade-offs. Siderhurst et al. (2025) present a low-cost harmonic radar (HR) system tailored to nest-finding. The approach is elegantly frugal—light, inexpensive tags and consumer transceivers—yet the HR’s reduced detection range and the inability to uniquely identify individuals highlight a recurring theme: inexpensive approaches can democratize methods but may require complementary tools or careful study design to overcome limitations. The responsible adoption of affordable methods, therefore, needs explicit reporting of ranges, biases, and recommended workarounds; several papers in this issue set a strong example by quantifying those boundaries.

## Non-Destructive Sampling, Museum Science, and Microbial Ecology

Brown et al. (2025) address a methodological conundrum that museum curators and molecular ecologists have long faced: how to extract DNA for genomic and microbiome work while preserving voucher morphology (Brown et al. 2025). Their Chelex-based, non-destructive protocol—with a pre-lysis bleach step and streamlined proteinase handling—produces high yields for both host and associated microbes while preserving diagnostic characters. This advance is consequential: it lowers the barrier for integrating historical collections into contemporary host-microbiome and phylogenomic studies, and thus links retrospective baselines with modern monitoring. The authors’ rigorous side-by-side comparisons with column methods and their explicit discussion of contamination control are the model best practice for museum molecular workflows.

## From Sequences to Diets: Metabarcoding’s Power and Pitfalls

Gay et al. (2025) exploit metabarcoding of hive matrices to compare the spring diets of honey bees and buff-tailed bumble bees. The biological result—generalist diets with substantial but marker-dependent overlap—is less the headline than their methodological lesson: marker choice (*ITS2* vs. *trnL* variants) dramatically influences taxon detection and inferred niche overlap. That finding is both sobering and actionable. For those using metabarcoding to map interactions, this article makes it clear: use multiple markers, expand reference libraries, and apply models that account for sample-type biases (honey versus wax pot content). Paired with Brown et al. (2025), the collection forges a pipeline connecting museum specimens, molecular assays, and robust ecological conclusions.

## Scaling Detection: Acoustics, Imaging, and Automated Pipelines

Automated detection is essential for scaling ecological monitoring. In Hearon et al. (2025), open-source CNN Buzzdetect achieves ∼95% precision on passive acoustic data and offers an accessible platform for large-scale pollinator surveys—but its lower sensitivity and bias toward some buzz types require validation with ground-truth data. Similarly, Faraji et al. (2025) show that relatively simple image-processing tools (ImageJ) can produce operationally robust counts when algorithms are transparent and well validated. Together, these studies point to a practical future in which modular, open-source detection tools—each with documented error profiles—are integrated into monitoring workflows to produce richer temporal and spatial datasets.

## Physiology, Climate, and Management Implications

Botsch et al. (2025) probe short-term heat exposure effects on western corn rootworm thermal tolerance and find that brief heat exposure accumulates stress rather than inducing protective hardening, that is, a hormetic response. This empirical result has immediate implications for pest management and modeling: temperature histories and transient heat events may reduce pest performance more than static thermal maxima predict. Its inclusion in a methods issue is apt—robust assays of *CTmax* and standardized heat-hardening protocols are needed to make physiology comparable across studies and useful for forecasting pest dynamics under climate variability.

## Synthesis: What This Collection Collectively Enables

Taken together, these articles build a connected toolkit. Non-destructive DNA extraction enables historical baselines; metabarcoding (if carefully chosen and validated) links diet to landscape; acoustic and image-based detection scale temporal monitoring; fluorophores and HR permit direct tracking and experimental manipulation; thermal assays ground mechanistic predictions about pest responses to climate. The common values are reproducibility (open code, standardized protocols), accessibility (low cost, off-the-shelf components), and explicit quantification of limitations. Those qualities make the methods both usable and improvable by the insect science community.

## Future Directions and Recommendations

1. Standardized benchmarks and inter-lab ring tests: several papers demonstrate method sensitivity to operator and ecological context. Coordinated benchmarking (ring tests) across labs and habitats will accelerate adoption and clarify boundaries of applicability.
2. Multi-method validation: since every approach has biases (eg marker choice in metabarcoding, sensitivity profiles in bioacoustics, detection distance in HR), pairing complementary methods in pilot studies should become routine.
3. Open data, models, and training sets: the value of tools such as Buzzdetect multiplies when labeled datasets are shared. Funders and journals should incentivize deposition of training audio, images, and raw assay data.
4. Bridging detection and function: detecting pollinator visits (acoustics) or presence (marks, tracking) is necessary but not sufficient for inferring services. Studies that pair methodological detection with functional assays (pollen transfer, seed set) are a priority.
5. Ethical and curatorial guidelines: non-destructive methods invite broader use of collections, but community guidelines are needed on acceptable treatments (eg bleach concentrations, resin exposure) to protect irreplaceable specimens.
6. Operational translation: for public health and pest management, co-development with end users (vector control districts, growers) will ensure that methods like ImageJ counting or HR tracking are adopted into decision frameworks.

## Conclusion

This Special Collection exemplifies how careful method development—attentive to field constraints, openly shared, and rigorously validated—multiplies scientific reach. The 7 contributions move insect science toward a future where measurements are more reliable, less destructive, and more widely available. Readers should not view these articles as final solutions but as well-documented starting points: adopt them, test them in your systems, and report back. Methodological progress in entomology will accelerate fastest when innovations are shared, stress-tested, and integrated across the scales captured in this issue.

## Data Availability

This editorial contains no original datasets. All data, studies, and resources discussed are cited and available in the public literature (see “References” section).
